# Factors Associated With Prioritizing Quick Firearm Access Over Suicide Prevention Among US Adults

**DOI:** 10.1111/sltb.70131

**Published:** 2026-07-28

**Authors:** Michael D. Anestis, Allison E. Bond

**Affiliations:** ^1^ New Jersey Gun Violence Research Center Piscataway New Jersey USA; ^2^ Rutgers School of Public Health Piscataway New Jersey USA

## Abstract

**Objective:**

To examine characteristics associated with US adults favoring storing firearms unlocked to ensure quick access in an emergency, even if doing so increases the risk for suicide.

**Method:**

A total of 2963 adults with home firearm access were drawn from a nationally representative sample of US adults (*n* = 7521). Participants were members of a probability‐based panel and completed a self‐report survey between June 24 and July 10, 2025.

**Results:**

Being female (OR = 0.82; 95% CI = 0.69–0.99), children in the home (OR = 0.41; 95% CI = 0.33–0.50), trust in the government for protection (OR = 0.89; 95% CI = 0.82–0.97), and beliefs that home firearm access increases suicide risk (OR = 0.66; 95% CI = 0.59–0.75) were associated with lower odds of endorsing firearms should be stored unlocked. Loaded and unlocked storage (OR = 5.90; 95% CI = 4.88–7.15), frequent firearm carrying (OR = 1.08; 95% CI = 1.01–1.15), and belief that firearms are essential for protection (OR = 1.40; 95% CI = 1.27–1.54) were associated with greater odds of endorsing firearms should be stored unlocked.

**Conclusions:**

The results could mean those who feel responsible for protecting loved ones and believe firearms are essential for that task are more likely to endorse favoring unlocked firearm storage, even if they believe this will increase suicide risk.

## 1 Introduction

Secure firearm storage protects against firearm injury and death (Grossman et al. 2005), yet the frequency with which US adults utilize secure storage is suboptimal (Anestis, Moceri‐Brooks, et al. 2023; Betz et al. 2023). One possibility—supported through both quantitative and qualitative research—is that this is driven by two factors: the tendency for home firearm access to be motivated by the drive for home protection and a general lack of belief in an association between firearm storage and suicide risk (Ye et al. 2022; Anestis et al. 2018; Aitken et al. 2020; Pallin et al. 2021; Barton and Kologi 2015; Ewell Foster et al. 2024). Results from a recent nationally representative survey (Anestis et al. 2025) indicated most adults believe firearm access helps protect household members during home invasions, and that many believe firearms are unrelated to suicide. In that survey, unsecure firearm storage was common even among those who believe firearm access is related to suicide. Given the latter finding, it appears unsecure storage may reflect a cost‐benefit analysis in which individuals weigh the likelihood and cost of various threats and conclude protection from external threats (e.g., home invasion) is more important. Which individuals would make this choice remains unclear. This study aimed to assess factors associated with believing it makes more sense to store firearms unlocked for quick access in an emergency, even if this increases suicide risk.

## 2 Methods

A nationally representative sample of US adults was collected via Ipsos' probability‐based KnowledgePanel June 24–July 10, 2025. Eligible adults (*n* = 13,623) were sent a link. A majority (*n* = 8190; 60%) opened the link, with 7521 (92%) providing informed consent and completing the survey. Only individuals who endorsed home firearm access (*n* = 2963; 39.4%) were included in these analyses. The final analytic sample, accounting for missingness, included 2873 individuals with household firearm access (Table 1). Data were weighted for representativeness. This study is consistent with AAPOR guidelines. Ethical approval was provided by the Rutgers Health institutional review board.

**TABLE 1:** Demographic composition of those who believe it makes more sense to store a firearm locked up to prevent suicide, even if it makes it more difficult to access in an emergency relative to those who believe it makes more sense to store a firearm unlocked for quick access in an emergency, even if it increases the risk that somebody in the home will die by suicide.

	Makes more sense to store a firearm…		*Χ* ^2^	*p*	Φ/V
	Locked up to prevent suicide	Unlocked for quick access in emergency			
	% (weighted n)	% (weighted n)			
Full sample	63.5 (1824)	36.5 (1049)			
Sex			47.36	< 0.001	−0.13
Male	48.1 (877)	61.4 (644)			
Female	51.9 (947)	38.6 (405)			
Race/Ethnicity			8.89	0.064	0.06
White, Non‐Hispanic	68.5 (1248)	73.3 (770)			
Black, Non‐Hispanic	10.6 (193)	9.7 (102)			
Other, Non‐Hispanic	4.9 (89)	3.4 (36)			
Hispanic	14.2 (258)	11.8 (124)			
2+ Races, Non‐Hispanic	1.9 (35)	1.7 (18)			
Kids in home			81.05	< 0.001	−0.17
Yes	36.4 (663)	20.3 (213)			
No	63.6 (1160)	79.7 (836)			
Unlocked & loaded firearms			565.70	< 0.001	0.45
Yes	15.6 (283)	58.3 (608)			
No	84.4 (1536)	41.7 (434)			

### 2.1 Measures

Demographic variables were assessed using items embedded in KnowledgePanel member profiles. These included sex, age, education, and presence of children in the home.

Political beliefs were assessed by asking participants “How would you characterize your political beliefs?” Answers ranged from 1 (“Highly conservative”) to 5 (“Highly liberal”).

Threat sensitivity was assessed using the Posttraumatic Cognitions Inventory (Foa et al. 1999). Because the measure was originally designed to assess symptoms of posttraumatic stress disorder, participants are generally asked to consider their responses within the context of a specific traumatic event. Consistent with prior research (Anestis, Bandel, et al. 2023), we removed the prompt asking participants to reference any specific traumatic event when providing their responses, instead asking them to reflect on the general degree to which they experience the world and other people as threatening.

Trust in the government for protection and the belief that firearms are essential for protection were assessed using items developed specifically for this protocol. Participants were told to “please indicate how well each of the following statements applies to you.” The items used for this analysis included “I trust the federal government to protect my personal freedoms and security” and “Guns are essential for protection from other citizens.”

To assess the belief that firearm access is associated with suicide risk, participants were asked to “please select the answer that you believe best reflects the truth.” The prompt for the item said “Keeping a firearm in or around the home” and answer choices included “is extremely helpful in preventing suicide,” “is somewhat helpful in preventing suicide,” “has no impact on the risk for suicide,” “somewhat increases the risk of suicide,” and “dramatically increases the risk for suicide.”

Firearm carrying was assessed by asking “how frequently do you carry a firearm on your person outside of your home?” Responses ranged from 0 (“Never”) to 5 (“Always”).

To assess firearm suicide loss status, participants were asked “Please indicate whether you have had each of the following experiences,” with one of the response items then saying “Have you ever known someone personally that has died by suicide with a firearm?” Response options included “Yes” and “No.”

Lifetime experience of a home invasion was assessed by asking participants “have you ever experienced a burglary, break‐in, or home invasion?” Response options included “Yes” and “No.”

The dependent variable asked which statement best aligns with how participants feel, even if neither perfectly represents the truth: (1) It makes more sense to store a firearm locked up to reduce the risk that someone in the home will die by suicide, even if this will make it take longer to get to in an emergency or (2) It makes more sense to store a firearm unlocked so you can get to it quickly in an emergency, even if this increases the risk that someone in the home might die by suicide.

### 2.2 Data Analytic Plan

To assess univariate differences in storage preference across demographic characteristics, we conducted a series of chi‐squared analyses. For demographic variables with two categories, phi served as the index of effect size. For demographic variables with three or more categories, Cramer's V served as the index of effect size.

To test our multivariate model examining factors associated with prioritizing unlocked firearm storage to facilitate quick access in an emergency, even if this increases the risk of suicide, we conducted a binary logistic regression. Given the lack of prior research on this specific outcome variable, the optimal list of variables to include was not easily determined via a review of prior literature. As such, we selected variables that reflected aspects of an individual's identity, belief system, or life experiences that we believed may influence their decision if forced to choose one of the two available options for firearm storage preferences. Demographic factors (sex, age, education, and presence of children in the home), political beliefs, threat sensitivity, trust in the government for protection, the belief that firearms are essential for protection from others, the belief that household firearm access is associated with suicide risk, the frequency of firearm carrying outside the home, firearm suicide loss survivor status, and lifetime experience of a home invasion served as the independent variables. Preference for locked (vs unlocked) firearm storage served as the dependent variable. Odds ratios served as the index of effect size. All analyses were run utilizing SPSS version 31.0.0.0.

## 3 Results

A majority (63.5%, *n* = 1824) indicated it makes more sense to store firearms locked to reduce suicide risk, even if it requires more time to access the firearm in an emergency. Among those that favored storing firearms unlocked, 58.3% endorsed typically storing at least one firearm loaded and unlocked (vs 15.6% of those who favored storing firearms locked; *Χ* ^2^ = 565.70; *p* < 0.001; Φ = 0.45). Among those who favored storing firearms unlocked, 61.4% identified as men (vs 48.1% of those who favored storing firearms locked; *Χ* ^2^ = 47.36; *p* < 0.001; Φ = −0.13) and 20.3% endorsed having children living in the home (vs 36.4% of those who favored storing firearms locked; *Χ* ^2^ = 81.05; *p* < 0.001; Φ = −0.17; Table 2).

**TABLE 2:** Logistic regression examining factors associated with greater or lesser odds of believing it makes more sense to store a firearm unlocked so you can get to it quickly in an emergency, even if this increases the risk that someone in the home might die by suicide (vs belief that it makes more sense to store a firearm locked up to reduce the risk that someone in the home will die by suicide, even if this will make it take longer to get to in an emergency).

Firearm storage risk preference—Protect from others vs protect from suicide	Wald	OR	*p*	95% CI
Gender (Ref: Male)	5.87	0.81	0.015	0.68–0.96
Age	2.39	1.00	0.122	0.99–1.00
Education	0.00	1.00	0.949	0.91–1.09
Political beliefs	27.02	0.79	< 0.001	0.72–0.86
Kids in home (Age 0–17)	102.46	0.34	< 0.001	0.28–0.42
Threat sensitivity	2.60	1.02	0.107	0.99–1.00
Firearm carrying frequency	38.81	1.22	< 0.001	1.14–1.29
Trust government to protect freedom & security	8.30	0.89	0.004	0.83–0.97
Guns are essential for protection	41.94	1.37	< 0.001	1.25–1.51
Home firearms and suicide risk (increased risk at higher score)	36.18	0.69	< 0.001	0.62–0.78
Known someone who died by firearm suicide	3.05	0.85	0.081	0.72–1.02
Experienced home invasion	2.13	1.15	0.144	0.95–1.39

*Note:* DV: 0 = Makes more sense to store a firearm locked up to reduce the risk that someone in the home will die by suicide, even if this will make it take longer to get to in an emergency. 1 = Makes more sense to store a firearm unlocked so you can get to it quickly in an emergency, even if this increases the risk that someone in the home might die by suicide. Sex, kids in home, firearm storage, firearm suicide loss survivor status, and home invasion experience were all scored dichotomously.

A logistic regression (Table 2) found being female (OR = 0.81; 95% CI = 0.68–0.96), endorsing more liberal political beliefs (OR = 0.79; 95% CI = 0.72–0.86), having children living in the home (OR = 0.34; 95% CI = 0.28–0.42), endorsing greater trust in the government for protection (OR = 0.89; 95% CI = 0.83–0.97), and a stronger belief that home firearm access increases suicide risk (OR = 0.69; 95% CI = 0.62–0.78) were associated with lower odds of endorsing firearms should be stored unlocked for quick access in an emergency even if this increases the risk of suicide. More frequent firearm carrying (OR = 1.22; 95% CI = 1.14–1.29) and a greater belief that firearms are essential for protection (OR = 1.37; 95% CI = 1.25–1.51) were associated with greater odds of endorsing firearms should be stored unlocked for quick access in an emergency even if this increases the risk of suicide.^1^

## 4 Discussion

Firearm owners often balance protection from external (e.g., home invasion) and internal threats (e.g., suicide). Our results demonstrate that, even when told doing so may increase suicide risk, many favor storing firearms unlocked to ensure quick access during an emergency. Our findings also indicate that, when firearm owners have children living in the home and believe firearm access is linked to suicide, they are less likely to prioritize quick access. In contrast, those who see firearms as vital for protection and who carry firearms more frequently are more likely to prioritize quick access, even if they know this increases suicide risk.

We interpret our results as indicating that those who feel responsible for protecting their families and who believe firearms are vital for performing that task prioritize protection from external threats over protection from threats within the home. Faced with a forced choice in which one threat must be prioritized over the other, such individuals have greater odds of indicating that protection from external threats should drive firearm storage decision making. This appears particularly true for men, highlighting the potential role of masculinity and honor culture (Bock et al. 2024) in firearm storage decisions.

### 4.1 Public Health Implications

From a prevention perspective, this highlights the importance of framing secure firearm storage as home security. Rather than arguing against the common drive for home protection, prevention efforts might consider leveraging this instinct, but reframing suicide prevention as a vital component of home protection, thereby recasting secure firearm storage as a behavior consistent with core values. Accomplishing this would require effective overt messaging by trusted messengers (Bond et al. 2022), as well as covert messaging such as embedding secure firearm storage into popular media (Brady United, n.d.).

Limitations include the use of cross‐sectional and self‐report data. We were also unable to assess degree of rurality, which limits our understanding of a potentially important component of neighborhood environment that could influence firearm storage decision making and preferences. Despite this, our findings provide increased clarity on factors associated with prioritizing quick firearm access even if they believe such access increases suicide risk.

## Author Contributions

**Michael D. Anestis:** conceptualization, funding acquisition, writing – original draft, methodology, writing – review and editing, formal analysis. **Allison E. Bond:** writing – original draft, writing – review and editing.

## Funding

This project was funded via the annual budget of the New Jersey Gun Violence Research Center, which is funded through the New Jersey Office of the Secretary of Higher Education.

## Ethics Statement

This project was approved by the Rutgers Health Institutional Review Board.

## Consent

All participants provided informed consent prior to participation.

## Conflicts of Interest

Dr. Anestis reports grant funding as well as personal income in the form of book royalties, speaking fees, consulting fees, and training fees related to secure firearm storage.

## Data Availability

Data can be made available upon request of the corresponding author.

## Endnotes

^1^
We ran two sets of sensitivity analyses. In the first, we only included participants who indicated that they consider the firearms in or around their home to be theirs. In the second, we only included participants who indicated that they make decisions regarding how firearms in or around the home are stored. In both cases, the results remained unchanged with respect to statistical significance with one exception: women did not differ from men in their storage preference in either sensitivity analysis. This likely reflects the shift in sex composition between the groups in the sensitivity analyses relative to the primary analysis (men were more heavily represented in the sensitivity analyses).
